# Resuscitative thoracotomy in traumatic cardiac arrest: Multisociety consensus recommendations for settings with a low prevalence of penetrating injuries

**DOI:** 10.1007/s00508-026-02771-3

**Published:** 2026-06-13

**Authors:** Martin W. Dünser, Daniel Grassmann, Thomas Hamp, Mario Krammel, Philipp Eller, Philip Eisenburger, Romana Erblich, Barbara Hallmann, Michael Halmich, Klaus Hellwagner, Klaus Herbich, Harald Herkner, Igor Knez, Lukas L. Negrin, Marcel Rigaud, Joachim Schlieber, Sebastian Schnaubelt, Wolfgang Schreiber, Alexandra-Maria Stommel, Florian Tomaselli, Helmut Trimmel, Christoph Veigl, Wolfgang Voelckel, Stefan Watzka, Dominik Wiedemann, Paul Puchwein

**Affiliations:** 1 Austrian Society of Anaesthesiology, Reanimatology and Critical Care Medicine (ÖGARI), Vienna, Austria; 2 Department of Anaesthesiology and Critical Care Medicine, Kepler University Hospital, Johannes Kepler University Linz, Krankenhausstrasse 9, 4020 Linz, Austria; 3 Federation of Austrian Societies of Intensive Care Medicine (FASIM), https://fasim.at/; 4 Emergency Medical Service Vienna (Berufsrettung Wien), Vienna, Austria; 5 Austrian Cardiac Arrest Awareness Association (PULS), Vienna, Austria; 6 Austrian Association of Emergency and Disaster Medicine (ÖNK), https://oenk.org/; 7 Austrian Society of Internal and General Intensive Care and Emergency Medicine (ÖGIAIN), https://www.intensivmedizin.at/; 8 Department of Internal Medicine, Medical University of Graz, Graz, Austria; 9 Department of Emergency Medicine and Internal Medicine, Klinik Floridsdorf, Vienna Healthcare Group, Vienna, Austria; 10 Working Group Emergency Medicine (AGN), https://www.agn.at/; 11 Department of Anaesthesiology and Intensive Care Medicine, Medical University of Graz, Graz, Austria; 12 Austrian Society of Ethics and Law in Emergency and Disaster Medicine (ÖGERN), https://www.oegern.at/; 13 7th Department of Internal Medicine with Emergency Medicine, Klinik Hietzing, Vienna Healthcare Group, Vienna, Austria; 14 ÖAMTC Flugrettung, Vienna, Austria; 15 Austrian Association of Emergency Medicine (AAEM), https://www.aaem.at/; 16 Department of Emergency Medicine, Medical University of Vienna, Vienna, Austria; 17 Austrian Society of Cardiac and Thoracic Vascular Surgery (ÖGHTG), https://www.oeghtg.at/; 18 Division of Cardiac Surgery, Department of Surgery, Medical University of Graz, Graz, Austria; 19 Austrian Society of Orthopaedics and Traumatology (ÖGOuT), https://www.oegout.at/; 20 Department of Orthopaedics and Trauma Surgery, Medical University of Vienna, Vienna, Austria; 21 Department of Anaesthesiology and Critical Care Medicine, AUVA Trauma Centre Graz, Graz, Austria; 22 Austrian Resuscitation Council (ARC), https://wiederbelebung.at/; 23 Department of Anaesthesiology and Intensive Care Medicine, AUVA Trauma Centre Salzburg, Salzburg, Austria; 24 Austrian Society of Thoracic Surgery (ÖGTC), https://ogtc.at/; 25 Department of Cardiac, Vascular and Thoracic Surgery, Johannes Kepler University Linz, Linz, Austria; 26 Karl Landsteiner Institute for Emergency Medicine and Patient Safety, Seebenstein, Austria; 27 Department of Anesthesiology and Intensive Care Medicine, University Hospital St. Pölten—NOE LGA, Karl Landsteiner University, St. Pölten, Austria; 28 Department of Thoracic Surgery, Klinik Floridsdorf, Vienna Healthcare Group, Vienna, Austria; 29 Department of Cardiac Surgery, University Hospital St. Pölten—NOE LGA, Karl Landsteiner University, St. Pölten, Austria; 30 Department of Orthopaedics and Traumatology, Medical University of Graz, Graz, Austria

**Keywords:** Traumatic cardiac arrest, Low prevalence, Penetrating injuries, Delphi, Emergency medical services

## Abstract

**Background:**

Resuscitative thoracotomy (RT) is recommended for selected patients with traumatic cardiac arrest, particularly after penetrating trauma; however, most evidence is derived from high-volume centers, creating uncertainty about its implementation in settings with a low prevalence of penetrating injuries. This study aimed to develop evidence-informed, consensus-based recommendations for the systematic implementation of RT in such contexts.

**Methods:**

A modified Delphi process was conducted with 24 specialists representing 10 Austrian medical societies and two emergency medical services. A systematic literature review was carried out to enable the development of 22 open-ended questions. Responses from the first round were synthesized into 28 statements.

**Results:**

Consensus was achieved on all 28 statements after 4 rounds, with participation rates of 100% in rounds 1 and 2, and 91.7% in rounds 3 and 4. The panel issued 1 strong recommendation, 15 weak recommendations and 12 best-practice statements. Agreement was unanimous in 17.9%, strong in 50.0%, and moderate in 32.1% of the statements. Evidence certainty was rated as moderate (18.8%), low (43.8%) or very low (37.5%). Key recommendations emphasized strict indication criteria, system level prerequisites such as major trauma centers and physician-led emergency services, preference for the clamshell technique and the need for structured multidisciplinary training and governance.

**Conclusion:**

This consensus provides guidance on implementing RT in settings with a low prevalence of penetrating trauma. Careful patient selection, system preparedness and structured training are essential to optimize outcomes. These recommendations can support clinicians and policymakers in their decision if and how RT should be implemented into trauma systems with limited exposure to penetrating injuries.

**Supplementary Information:**

The online version of this article (10.1007/s00508-026-02771-3) contains supplementary material, which is available to authorized users.

## Background

Traumatic cardiac arrest (TCA) predominantly affects young, previously healthy individuals and is associated with high mortality rates [1]. A resuscitative thoracotomy (RT) is a potentially life-saving emergency surgical procedure recommended for selected TCA patients, particularly those following penetrating chest trauma [2–5]. The European Resuscitation Council (ERC) 2025 guidelines recommend RT for patients with recent (< 15 min) TCA due to cardiac tamponade following penetrating chest injury or for proximal vascular control; however, these guidelines specify that RT should only proceed within 15 min of TCA if appropriate expertise, environment and equipment are available [6]. In doing so, the ERC is the first to emphasize that RT requires specific institutional prerequisites, including system preparedness, specialized equipment and targeted education [6]. While these requirements are often met in regions where penetrating injuries are prevalent, they may be lacking in systems where such trauma is rare or respective numbers are rising.

Austria is a country with an overall low prevalence of penetrating injuries [7, 8], although incidences of weapon-related violence have recently increased [9]. A retrospective cohort study identified an incidence of 3.6 penetrating chest injuries per 100,000 inhabitants in the capital city of Vienna in 2017, translating to approximately one case per week [8]. Nonetheless, TCA from penetrating trauma remains a rare clinical scenario in Austria. Many emergency physicians and trauma surgeons are rarely, if ever, exposed to such pathologies during their careers. As a result, most Austrian trauma teams are currently neither prepared to perform RT nor to provide the necessary postprocedural management. Moral, personal, and systemic conflicts appear inevitable when guidelines recommend RT to save a life, yet emergency medical services (EMS) or hospital trauma teams lack the knowledge and infrastructure to deliver it and no national guidance on system preparedness exists.

Given that established guidelines and the majority of evidence regarding RT originate from high-volume settings, there is limited scientific direction on if and how RT should be systematically implemented in low-prevalence environments. In this context, an evidence-informed expert consensus might represent the best approach to providing recommendations on system requirements, indications, technical aspects, training and education, quality management and the ethical and legal considerations of implementing RT in countries with a low prevalence of penetrating injuries, such as Austria.

## Methods

### Objective

This research project was designed as a modified Delphi study and aimed to produce evidence-informed, consensus-based recommendations on the infrastructure and requirements necessary to systematically implement RT in a low-prevalence setting for penetrating trauma, such as Austria. Furthermore, it sought to provide comprehensive guidance on indications, practical application, prehospital considerations and continuing education and training. The project also addressed quality management as well as ethical and legal considerations for healthcare systems aiming to systematically incorporate RT into the clinical practice. Prior to commencing the Delphi process, the study protocol was registered on the Open Science Framework (OSF; https://osf.io/a6hd8/) on 19 October 2025 and updated on 25 October 2025.

### Steering committee and expert panel selection

The steering committee comprised four persons (MWD, MK, DG, PP) representing the Austrian Society of Anesthesiology, Resuscitation and Critical Care Medicine (ÖGARI), the Emergency Medical Service Vienna (EMS Vienna), the Austrian Association of Emergency and Disaster Medicine (ÖNK), as well as the Austrian Society of Orthopedics and Traumatology (ÖGOuT). All members of the steering committee possess expertise in the field of RT, ranging from clinical application, simulation-based training and education to quality management. Subsequently, the presidents of all Austrian societies involved in emergency medicine, resuscitation, traumatology, cardiac surgery and thoracic surgery were invited to nominate two representatives with specialized knowledge, interest and/or experience in RT. Furthermore, the chief medical officers of the EMS Vienna and the helicopter EMS ÖAMTC Flugrettung were contacted to nominate two representatives based on the same criteria. The inclusion of the EMS Vienna was motivated by its role as the first service in Austria to systematically implement prehospital RT. Similarly, the ÖAMTC Flugrettung was included as the nation’s largest helicopter EMS, providing high-level prehospital care across the entire country. Given the invasiveness of the procedure and the extreme circumstances under which it is performed, particularly in low-prevalence settings, the steering committee deliberately decided not to involve members of the general public in the Delphi process.

The final expert panel consisted of 24 specialists representing 10 professional medical societies and two major prehospital emergency medical services (listed in alphabetical order): Austrian Association of Emergency Medicine (AAEM); Arbeitsgemeinschaft Notfallmedizin (AGN)—Working Group Emergency Medicine; Austrian Resuscitation Council (ARC); Berufsrettung Wien (MA 70)—EMS Vienna; ÖAMTC Flugrettung—Austrian Automobile, Motorbike and Touring Club Air Rescue ; Österreichische Gesellschaft für Anästhesie, Reanimation und Intensivmedizin (ÖGARI)—Austrian Society of Anesthesiology, Resuscitation and Critical Care Medicine; Österreichische Gesellschaft für Ethik und Recht in der Notfall- und Katastrophenmedizin (ÖGERN)—Austrian Society for Ethics and Law in Emergency and Disaster Medicine; Österreichische Gesellschaft für Herz- und thorakale Gefäßchirurgie (ÖGHTG)—Austrian Society of Cardiac and Thoracic Vascular Surgery; Österreichische Gesellschaft für internistische und Allgemeine Intensivmedizin und Notfallmedizin (ÖGIAIN)—Austrian Society of Medical and General Critical Care and Emergency Medicine; Österreichische Gesellschaft für Orthopädie und Traumatologie (ÖGOuT)—Austrian Society of Orthopedics and Traumatology; Österreichische Gesellschaft für Thoraxchirurgie (ÖGTC)—Austrian Society of Thoracic Surgery; Österreichische Gesellschaft für Notfall- und Katastrophenmedizin (ÖNK)—Austrian Society of Emergency and Disaster Medicine.

### Definition of resuscitative thoracotomy

This consensus statement defines RT as an emergency surgical procedure to access the thoracic cavity in a patient with TCA for whom resuscitative efforts are indicated. It specifically addresses RT performed outside of an operating theater by physicians who are not specialized in cardiac or thoracic surgery.

### Systematic review of the literature

To provide the expert panel with a comprehensive overview of the contemporary scientific evidence, a systematic literature review was conducted. The details of this literature review are outlined in Electronic Supplementary Material Table 1. The MEDLINE database was searched via PubMed® (https://pubmed.ncbi.nlm.nih.gov) using predefined search terms, primarily focusing on identifying original studies as well as national and international guidelines. Additionally, bibliographic references and citations from the included studies and relevant review articles were screened to identify further potentially eligible publications. Research articles published between 1 January 1966 and 30 September 2025 were reviewed. The search was subsequently updated to extend its coverage until 28 February 2026. Study selection was limited to publications in English and two researchers (RE, CV) independently screened all records. In the first stage, titles and abstracts were screened for relevance; then, full-text versions of all potentially eligible studies were retrieved. These full-texts, along with an abstract-based summary of the evidence, were made available to all Delphi panellists via a dedicated online platform (Dropbox®; Dropbox Inc., San Francisco, CA, USA). A summary of the results of the literature search is provided in Electronic Supplementary Material Fig. 1.

### Development of open-ended questions to be answered by the expert panel

Based on the results of the systematic literature review, the steering committee drafted a preliminary set of questions aligned with the study objectives. Subsequently, an online meeting was held with the expert panel to discuss, expand and refine these items. During this session, the methodology and details of the Delphi process were explained to all panel members. Following this collaborative review, a final set of 22 questions was approved by the steering committee, to be answered by the expert panel. These questions were categorized into seven thematic blocks: system requirements for implementing RT, indications for RT, practical implementation of RT, special considerations for the prehospital setting, education and training, quality management, ethical and legal considerations. The complete questionnaire is detailed in Electronic Supplementary Table 2.

### Design and conduct of the Delphi study

The design and conduct of this modified Delphi study were informed by established guidelines on designing, conducting and reporting Delphi processes [10, 11]. The final set of questions was communicated to the panellists 2 weeks prior to the start of the online process. An anonymous online survey platform (Survey Monkey®; SurveyMonkey Inc., San Mateo, CA, USA) was used for data collection. In the first round, all panellists including the members of the steering committee, were asked to provide answers to the open-ended questions. Each panellist was encouraged to provide multiple statements per question where appropriate. Following the completion of the first Delphi round, the steering committee synthesized these responses into 28 distinct statements. The certainty of evidence supporting each statement was rated as high, moderate, low or very low, according to the GRADE system [12]. This classification defined the strength of each recommendation, categorized as either strong (“we recommend”) or weak (“we suggest”). In instances where published evidence was insufficient or unavailable, “best practice statements” were formulated based on expert consensus.

In subsequent Delphi rounds, panellists rated their level of agreement with each statement using a 9-point Likert scale, where 1 represents “complete disagreement” and 9 represents “full agreement”. A score of 7 or higher was predefined as agreement with a statement. Group consensus was defined as at least 80% agreement among all panellists. The degree of consensus was further categorized as moderate (80–89.9%), strong (90–99.9%), or unanimous (100%). In addition to the Likert scale ratings, panellists were encouraged to provide qualitative comments on the wording of each statement. Statements that reached the consensus threshold were accepted and therefore removed from subsequent rounds. For items that did not reach consensus, the steering committee adjusted the wording based on the expert panel’s feedback before the next voting round. This iterative process continued until group consensus was achieved for all 28 statements. Prior to voting in each subsequent round, all panellists were provided with a summary of the Likert scale results from previous rounds and suggested refinements for each statement.

No financial or non-financial incentives were provided to encourage participation in the consensus process. Prior to the commencement of the second Delphi round, all panellists were reminded of the principles of cooperative behavior and the primary objective of the Delphi process: to achieve a robust group consensus rather than absolute individual agreement. It was emphasized that the iterative nature of the process might require participants to reconsider their initial position in light of the aggregate group feedback to facilitate a collective recommendation.

### Conflict of interest management

All panellists provided a written declaration of conflicts of interest prior to commencing the Delphi process. Potential conflicts of interests of single panellists are detailed in the respective section at the end of this manuscript. No panellist declared a conflict of interest that could have compromised unbiased decision-making or the integrity of the consensus recommendations.

## Results

The anonymous online Delphi process was initiated among the 24 panellists on 3 November 2025 and 4 rounds were conducted until group consensus was achieved for all 28 statements addressing the 22 study questions. Response rates remained high throughout the process, with 100% (24/24) participation in rounds 1 and 2 and 91.7% (22/24) in rounds 3 and 4. The process was formally concluded on 22 February 2026. Tables 1 and 2 summarize the final consensus statements alongside the respective degrees of agreement. The expert panel generated 1 strong recommendation, 15 weak recommendations and 12 best-practice statements. For the 16 recommendations supported by graded evidence (non-best practice), the certainty of evidence was moderate for 3 (18.8%), low for 7 (43.8%) and very low for 6 (37.5%) statements. Regarding the strength of consensus, unanimous agreement was reached for 5 statements (17.9%), strong agreement for 14 (50.0%), and moderate agreement for 9 (32.1%).

**Table 1:** Consensus statement on system requirements for implementing RT in a country with a low prevalence of penetrating injuries

Statement	Recommendation strength	Quality of evidence	Number of voting rounds to achieve consensus	Overall agreement (%)	Degree of agreement
*We recommend that systematic implementation of RT should only be considered in healthcare systems that fulfil all of the following criteria:*	Best practice statement	n. a.	1	91.7	Strong
A relevant caseload of patients with penetrating injuries within a geographic region accessible by EMS within 15 min					
A major trauma center with 24/7 thoracic or cardiac surgical capabilities					
A structured, physician-led prehospital EMS					
Availability of sufficient human, material, and financial resources to implement, maintain, and provide quality assurance for the practice of RT					

*EMS* emergency medical services, *n.* *a.* not applicable, *RT* resuscitative thoracotomy

**Table 2:** Consensus statements on the practice of RT for systems meeting requirements for implementing RT in a country with a low prevalence of penetrating injuries

Statements	Recommendation strength	Quality of evidence	Number of voting rounds to achieve consensus	Overall agreement (%)	Degree of agreement
**1. Indications for resuscitative thoracotomy**					
*1.1. What are the indications to perform RT?*					
We suggest performing RT on patients with TCA following penetrating trauma to the cardiac box if signs of life are present or if the duration of cardiac arrest is < 15 min	Weak	Moderate	1	95.7	Strong
We suggest performing RT on patients with TCA following blunt trauma and ultrasound-confirmed cardiac tamponade if signs of life or an organized ECG rhythm are present, or if the duration of cardiac arrest is < 10 min	Weak	Moderate	3	81.8	Moderate
*1.2. Does age or functional status influence the indication to perform RT?*					
We suggest applying the same indications for RT in children as in adults	Weak	Low	1	82.6	Moderate
We suggest against establishing a strict upper age limit for RT; however, we suggest that RT should not be performed in patients with multimorbidity or known life-limiting underlying conditions	Weak	Very low	1	95.7	Strong
*1.3. Is point-of-care ultrasound required before performing RT?*					
We suggest against the use of point-of-care ultrasound prior to performing RT in patients with TCA following penetrating trauma to the cardiac box who present with signs of life or a duration of cardiac arrest < 15 min	Weak	Low	1	87.0	Moderate
We suggest the use of point-of-care ultrasound to identify cardiac tamponade and indicate RT on patients with TCA following blunt trauma who present with signs of life, an organized ECG rhythm, or a duration of cardiac arrest < 10 min	Weak	Low	1	95.7	Strong
*1.4. Should RT also be performed in pregnant women?*					
We suggest that RT should be performed in pregnant women with TCA, provided that the indications for the procedure are met	Weak	Very low	1	91.3	Strong
*1.5. Which factors need to be considered when indicating RT in the case of multiple casualties?*					
We suggest against categorically withholding RT in the event of multiple casualties; however, we recommend a critical evaluation in eligible patients, ensuring that sufficient human and material resources are available in both the prehospital and hospital settings to support the procedure and subsequent care	Best practice statement	n. a.	1	87.0	Moderate
**2. Practical implementation of RT**					
*2.1. Which human resources are required to perform RT?*					
We suggest a minimum of 4–6 healthcare providers to perform RT. This team should include at least one emergency physician trained in the procedure and at least one provider proficient in advanced airway management	Weak	Very low	2	90.9	Strong
*2.2. Who should perform RT in the absence of a thoracic or cardiac surgeon?*					
We recommend that RT should only be performed by specialized physicians who are experienced in emergency medicine and specifically trained in this procedure	Best practice statement	n. a.	1	95.7	Strong
*2.3. Which technique should be used to perform RT?*					
We suggest using the clamshell technique as the preferred approach for performing RT	Weak	Moderate	1	87.0	Moderate
*2.4. Is pericardiocentesis an alternative to RT in patients with traumatic cardiac tamponade?*					
We suggest that pericardiocentesis should be reserved for patients with traumatic cardiac tamponade only when RT cannot be performed. This suggestion only applies if the required equipment and clinical expertise to perform this procedure are present	Weak	Low	2	86.4	Moderate
*2.5. Which requirements must the material for performing RT meet?*					
We suggest that the surgical instrumentation for RT should be standardized, streamlined, robust and user-friendly. Ideally, all necessary components should be prepacked in a compact, purpose-built thoracotomy kit	Weak	Very low	1	91.3	Strong
**3. Special considerations when performing RT in the prehospital setting**					
*3.1. Which specific safety measures should be taken into account when performing RT on scene?*					
As penetrating injuries are often caused by violent crimes, we suggest that RT should only be performed once scene safety has been ascertained	Best practice statement	n. a.	2	100	Unanimous
We suggest ensuring patient privacy by using visual shielding (e.g., privacy screens) and controlling scene access during the procedure	Best practice statement	n. a.	1	87.0	Moderate
*3.2. Are blood products required to perform RT in the prehospital setting?*					
We suggest that blood products should be available in prehospital systems performing RT; however, their immediate availability is not a prerequisite to perform RT in the prehospital setting	Weak	Very low	2	86.4	Moderate
*3.3. Which requirements should the receiving hospital meet to provide care for patients after RT in the prehospital setting?*					
We recommend that hospitals receiving patients following prehospital RT must be major trauma centers with 24/7 thoracic or cardiac surgical capabilities	Best practice statement	n. a.	1	95.7	Strong
We suggest that both the receiving hospital and the EMS performing prehospital RT adhere to coordinated treatment and communication protocols	Best practice statement	n. a.	1	95.7	Strong
*3.4. How should the emergency control centre be involved in the operational process of RT in the prehospital setting?*					
We suggest that the emergency control center serves as a critical link in the operational process of prehospital RT. Its role includes identifying eligible patients, rapidly dispatching and coordinating specialized resources and facilitating early communication with the receiving hospital	Best practice statement	n. a.	1	91.3	Strong
*3.5. Should other emergency services be informed or trained about the RT process?*					
We suggest that other emergency organizations, particularly the police, should be briefed on the operational requirements of prehospital RT and encouraged to participate in joint simulations to optimize scene management and interagency coordination	Best practice statement	n. a.	1	82.6	Moderate
**4. Education and training**					
*4.1. Which education and training are required to perform RT?*					
We suggest that physicians undergo a structured educational course including both cadaveric and multidisciplinary simulation training prior to performing RT	Weak	Low	1	95.7	Strong
We suggest that physicians trained in RT participate in periodic refresher training to maintain procedural skills and team coordination	Weak	Low	1	100	Unanimous
*4.2. Should nonphysician healthcare professionals involved in the process of RT also undergo education and training?*					
We suggest that nonphysician healthcare professionals involved in the RT process should also undergo structured education and training. This training should focus on procedural assistance, equipment familiarity and coordinated team dynamics during RT	Best practice statement	n. a.	1	100	Unanimous
**5. Quality management**					
*5.1. Which quality control measures should be taken when implementing RT?*					
We suggest that all systems performing RT implement comprehensive quality control and clinical governance measures. These should include mandatory structured case debriefings, regular clinical audits, and established feedback loops to ensure the ongoing refinement of protocols and training programs	Weak	Very low	1	100	Unanimous
*5.2. Which support options should be offered to healthcare professionals who performed or witnessed RT?*					
We suggest that systems performing RT provide easily accessible, low-threshold support mechanisms (e.g., peer support programs, professional psychological counselling) for all healthcare professionals who performed or witnessed RT	Best practice statement	n. a.	1	100	Unanimous
**6. Ethical and legal considerations**					
*6.1. Which ethical considerations should be taken into account when deciding on the indications for and performing RT?*					
We recommend that ethical decision-making must guide the indication of RT. This process must incorporate an objective assessment of survival probability, strict adherence to eligibility criteria, and a profound respect for patient dignity. Furthermore, it requires a balanced approach to resource allocation, transparent communication with families, and sensitivity to the emotional impact on both relatives and the healthcare team	Strong	Low	1	95.7	Strong
*6.2. Which legal considerations should be taken into account when performing RT?*					
We recommend that physicians performing RT adhere to the established legal framework and professional statutes governing life-saving emergency procedures in their respective jurisdiction	Best practice statement	n. a.	1	95.7	Strong

*EMS* emergency medical services, *n.* *a.* not applicable, *RT* resuscitative thoracotomy, *TCA* traumatic cardiac arrest, *ECG* electrocardiography

## System requirements for implementing RT

### Which requirements must healthcare systems meet to systematically implement RT?

We recommend that systematic implementation of RT should only be considered in healthcare systems that fulfil all of the following criteria:

- A relevant caseload of patients with penetrating injuries within a geographic region accessible by EMS within 15 min.
- A major trauma center with 24/7 thoracic or cardiac surgical capabilities.
- A structured, physician-led prehospital EMS.
- Availability of sufficient human, material and financial resources to implement, maintain and provide quality assurance for the practice of RT.

(Best practice statement)

#### Rationale

As no scientific evidence regarding the systemic requirements for implementing RT was identified, the expert panel based its recommendations on the characteristics of healthcare systems where RT has been successfully established [13–15]. Accordingly, the panellists agreed that both surgical and critical care capacities, necessary to perform RT and provide specialized postprocedural care, are fundamental requirements. In Austria, these requirements are typically met by major trauma or tertiary care centers. It is important to emphasize that the required 24/7 availability of thoracic or cardiac surgical expertise is not synonymous with the presence of a department of thoracic or cardiac surgery but refers more to the round-the-clock availability of surgeons experienced in managing trauma cases in these surgical fields.

Furthermore, the availability of human, material and financial resources is essential for implementing, maintaining and ensuring quality assurance for RT. Given the time sensitivity of TCA and the fact that most TCAs occur before hospital admission [16], the panel identified structured, physician-led prehospital EMS as a core requirement. As reported in several European metropolitan regions [16], physician-staffed EMS can reach TCA patients in a timely manner and bring expertise to perform RT in the field. For patients in an out-of-hospital TCA setting, on-scene RT is likely to provide a survival benefit over intra-arrest transport to an emergency department (ED), where RT would be subject to further delays [16, 17]. A final yet fundamental requirement, particularly in regions with a low prevalence of penetrating trauma, is a relevant caseload of penetrating injuries within a geographic area accessible by EMS within 15 min. The panel intentionally used the term “relevant” rather than a specific numerical threshold to denote a caseload sufficient to maintain clinical skills and staff experience. This minimum volume may vary by center and EMS system, depending on staffing levels and overall trauma volume. For instance, a registry analysis from a Norwegian trauma center reported a 19% survival rate for RT following the structured implementation of team and procedural training, despite only 26 RTs being performed over a 13-year period [18, 19].

## Recommendations for healthcare systems fulfilling these requirements and aiming to systematically implement RT into the practice

### 1. Indications for RT

#### 1.1. What are the indications to perform RT?

We suggest performing RT on patients with TCA following penetrating trauma to the cardiac box if signs of life are present or if the duration of cardiac arrest is < 15 min.

(Weak recommendation, moderate-quality evidence)

We suggest performing RT on patients with TCA following blunt trauma and ultrasound-confirmed cardiac tamponade if signs of life or an organized ECG rhythm are present or if the duration of cardiac arrest is < 10 min.

(Weak recommendation, moderate quality evidence)

##### Rationale

Although no randomized controlled trials have evaluated the impact of RT on outcomes in TCA, retrospective cohort studies, registry analyses and systematic reviews suggest that RT improves survival in TCA caused by cardiac tamponade following penetrating injuries [16, 20]. Depending on the mechanism (stab vs. gunshot wounds), large database analyses indicate that penetrating injuries to the cardiac box (defined as the area bordered laterally by the two midclavicular or nipple lines, cranially by the clavicular line and caudally by the costal margins) resulting in TCA are associated with an approximately 60% incidence of cardiac tamponade [21]. In contrast, TCA following blunt trauma is rarely caused by cardiac tamponade [22]; however, RT can still lead to survival in highly selected blunt trauma patients where tamponade is the underlying cause [16, 23]. When TCA in blunt trauma originates from mechanisms other than cardiac tamponade (e.g., exsanguination), the current literature suggests that RT does not meaningfully improve survival; in such cases, other interventions, such as resuscitative endovascular balloon occlusion of the aorta (REBOA), can be superior for proximal vascular control [24–26]. The likelihood of survival for patients undergoing RT further depends on the presence of positive clinical predictors and the duration of cardiac arrest. The presence of signs of life (e.g., agonal gasping, residual brainstem reflexes, spontaneous movements) before RT is a strong predictor of survival [27–29]. Furthermore, the elapsed time since the onset of TCA significantly influences the chances of neurologically intact recovery, with 10–15 min representing a critical window [13, 16, 21, 30]. The ECG rhythm at the time of RT serves as a surrogate marker for the duration of arrest and is thus a potent predictor of survival [16, 21]. Specifically, an organized ECG rhythm is associated with significantly higher survival rates compared to asystole or agonal rhythms. In TCA patients with cardiac tamponade, survival to hospital discharge has been reported at 48.3% for those presenting with pulseless electrical activity versus 12.3% for those in asystole or agonal rhythm at the time of RT [16]. Given the conflicting data on survival following RT in blunt trauma [30, 31] and in alignment with prospective multicentric cohort data [32], the panel chose to limit the recommended duration of TCA before RT in blunt trauma patients to 10 min if signs of life or an organized ECG rhythm are absent.

#### 1.2. Does age or functional status influence the indication to perform RT?

We suggest applying the same indications for RT in children as in adults.

(Weak recommendation, low-quality evidence)

We suggest against establishing a strict upper age limit for RT; however, we suggest that RT should not be performed in patients with multimorbidity or known life-limiting underlying conditions.

(Weak recommendation, very low-quality evidence)

##### Rationale

Substantially less scientific evidence exists regarding the role of RT in pediatric patients compared to adults with TCA [33]. A recent scoping review indicated that survival following pediatric RT was highest among older children and adolescents, with limited data supporting its utility in infants and younger children [33]. National database analyses revealed high mortality rates after pediatric RT, suggesting that the presence of signs of life prior to RT may be particularly relevant to a beneficial outcome, regardless of the injury mechanism [34]. Consequently, recent systematic review and practice management guidelines recommended indications for RT in children similar to those in adults [35]. Even less evidence exists regarding a potential upper age limit for RT. Although one trauma registry analysis demonstrated that survival after RT declined with age, reporting no survivors older than 57 years [36], other data indicate that chronological age is not an independent predictor of outcome [37]. Therefore, the panel decided against suggesting a specific upper age limit; however, supported by data from both nontraumatic and TCA [38–40] the panel emphasizes that RT should not be performed on patients with significant multimorbidity, frailty or known life-limiting underlying conditions.

#### 1.3. Is point-of-care ultrasound required before performing RT?

We suggest against the use of point-of-care ultrasound prior to performing RT in patients with TCA following penetrating trauma to the cardiac box who present with signs of life or a duration of cardiac arrest < 15 min.

(Weak recommendation, low-quality evidence)

We suggest the use of point-of-care ultrasound to identify cardiac tamponade and indicate RT on patients with TCA following blunt trauma who present with signs of life, an organized ECG rhythm or a duration of cardiac arrest < 10 min.

(Weak recommendation, low-quality evidence)

##### Rationale

Point-of-care ultrasound can reliably identify cardiac tamponade in patients with cardiac arrest [41]; however, conducting intra-arrest ultrasound requires specific resources, skills, and, most critically, time [42]. Given the high probability of cardiac tamponade in patients sustaining TCA following penetrating injury to the cardiac box (approximately 60%) [21], the panel suggests against the use of point-of-care ultrasound prior to RT in these patients, provided that signs of life are present or the duration of cardiac arrest is less than 15 min. In these scenarios, the delay associated with ultrasound may outweigh its diagnostic benefit. Conversely, as RT primarily offers a survival benefit in the rare instances where blunt trauma-induced TCA is caused by cardiac tamponade [16, 23], the use of point-of-care ultrasound is justified before proceeding with RT in blunt trauma patients, provided that there are signs of life or an organized ECG rhythm or the arrest duration is under 10 min. In a prospective observational study from a major US trauma center, point-of-care ultrasound (i.e., focused assessment using sonography for trauma) demonstrated high sensitivity in predicting RT survivors among patients in TCA. Notably, the likelihood of survival was nonexistent if both cardiac tamponade and cardiac motion were absent on initial ultrasound [43].

#### 1.4. Should RT also be performed in pregnant women?

We suggest that RT should be performed in pregnant women with TCA, provided that the indications for the procedure are met.

(Weak recommendation, very low-quality evidence)

##### Rationale

Compared to the nonpregnant population, pregnant women are nearly twice as likely to die following trauma and have a twofold higher risk of experiencing intentional or violent trauma [44]. While penetrating trauma accounts for only a minority of injuries during pregnancy, it is more likely than blunt trauma to result in catastrophic injuries, such as cardiac tamponade or massive hemorrhage, that precipitate TCA [45]. There is a significant paucity of evidence specifically addressing the indications for and outcomes of RT in pregnant women; however, successful maternal survival following RT for TCA due to penetrating chest injuries has been documented in the literature [46].

#### 1.5. Which factors need to be considered when indicating RT in cases of multiple casualties?

We suggest against categorically withholding RT in the event of multiple casualties; however, we recommend a critical evaluation in eligible patients, ensuring that sufficient human and material resources are available in both the prehospital and hospital settings to support the procedure and subsequent care.

(Best practice statement)

##### Rationale

Penetrating trauma, particularly from firearms, represents a primary mechanism of injury in mass casualty incidents. This remains a significant consideration even in countries with a low baseline prevalence of penetrating trauma, such as Austria [47]. A retrospective autopsy study indicated that the rate of potentially preventable deaths following civilian mass shooting events is substantial, often involving chest wounds that might have been survived with rapid intervention [48]. Although these data suggest a potential role for RT in mass casualty incidents involving penetrating trauma, the literature lacks documented cases of its systematic use in such scenarios. Consequently, the panel based its recommendation against categorically withholding RT on established principles of trauma triage and management [49]. The panel emphasizes that while RT should not be reflexively ruled out, its application requires a rigorous, case by case evaluation. This decision must account for the individual patient’s eligibility and, crucially, the availability of sufficient human and material resources in both prehospital and hospital settings to ensure that the care of other victims is not compromised.

### 2. Practical implementation of RT

#### 2.1. Which human resources are required to perform RT?

We suggest a minimum of 4–6 healthcare providers to perform RT. This team should include at least one emergency physician trained in the procedure and at least one provider proficient in advanced airway management.

(Weak recommendation, very low-quality evidence)

##### Rationale

No direct scientific evidence specifically addresses the optimal team size for RT. Rather than prescribing a fixed minimum number of personnel, current guidelines, such as those from the ERC, emphasize ensuring sufficient human resources to perform essential tasks simultaneously during both inhospital and out-of-hospital cardiac arrest [50]. Simulation and registry studies indicate that teams comprising four or more providers achieve higher chest compression fraction, faster advanced life support interventions and improved overall teamwork [51]. Furthermore, the presence of at least seven healthcare providers on scene has been associated with the highest odds of survival to hospital discharge following out-of-hospital cardiac arrest [52]. Given the extreme complexity of both the clinical scenario and the RT procedure itself, the panel suggests a minimum of 4–6 healthcare providers. This team should include at least one emergency physician trained in the procedure and one provider proficient in advanced airway management. The panel acknowledges that this represents a baseline requirement; while larger teams may facilitate superior task distribution and procedural coordination, achieving even this minimum staffing level can be challenging for emergency medical services (EMS) operating on scene. In a hospital setting, however, meeting these requirements is typically ensured by established trauma team protocols.

#### 2.2. Who should perform RT in the absence of a thoracic or cardiac surgeon?

We recommend that RT should only be performed by specialized physicians who are experienced in emergency medicine and specifically trained in this procedure.

(Best practice statement)

##### Rationale

To date, no study has specifically evaluated the relationship between specialty training of the performing physician and RT outcomes; however, data from healthcare systems that have successfully incorporated RT into clinical practice [13, 14] indicate that the majority of in-hospital RTs are performed by trauma surgeons or emergency physicians. Prehospital RT is almost exclusively performed by emergency physicians with advanced training [15, 16]. In these established systems, the performing physicians are typically highly experienced in emergency trauma care and have undergone rigorous, procedure-specific training. Furthermore, the ERC emphasizes that RT should be performed only by healthcare providers with sufficient expertise and adequate specialized training [6]. Consequently, the panel concluded that the operator’s specific proficiency and procedural training are more critical for successful implementation and patient safety than their primary medical speciality alone.

#### 2.3. Which technique should be used to perform RT?

We suggest using the clamshell technique as the preferred approach for performing RT.

(Weak recommendation, moderate-quality evidence)

##### Rationale

Several studies investigated the optimal technique for performing RT in patients with TCA. A study evaluating six RT incision techniques concluded that the clamshell incision provides the fastest and most definitive access to all thoracic structures for assessment and control. While the right and left anterolateral incisions can be successfully employed by surgeons with extensive experience in RT, the clamshell approach was found to be superior for general emergency use [53]. These findings were corroborated by a perfused human cadaver simulation, which reported that the time to control a standardized cardiac stab wound was significantly shorter for inexperienced surgical trainees using the clamshell approach compared to the left anterolateral approach [54]. Furthermore, two randomized controlled trials, one using human cadavers and another a living swine model, identified the clamshell technique (as developed by Barts Health NHS Trust clinicians at London’s Air Ambulance) as the ideal method for emergency physicians performing RT in the absence of a surgical specialist [55, 56]. Additionally, a retrospective study highlighted that the clamshell technique facilitates atrial appendage cannulation, providing a quick and reliable technique for achieving central vascular access to enhance resuscitation during TCA [57]. Given these advantages in exposure, speed, and procedural versatility, the panel recommends the clamshell techniques as the standard approach for RT.

#### 2.4. Is pericardiocentesis an alternative to RT in patients with traumatic cardiac tamponade?

We suggest that pericardiocentesis should be reserved for patients with traumatic cardiac tamponade only when RT cannot be performed. This suggestion only applies if the required equipment and clinical expertise to perform this procedure are present.

(Weak recommendation, low-quality evidence)

##### Rationale

Pericardiocentesis can effectively drain fluid from the pericardial sac and relieve intrapericardial pressure [58]. In hemodynamically stable patients, it may even serve as a definitive management option for traumatic cardiac tamponade [58], particularly when surgical intervention is unavailable or delayed, as demonstrated in case series from resource-limited environments [59]; however, pericardiocentesis does not facilitate the closure of cardiac wounds or the drainage of clotted blood (hemopericardium), which is a hallmark of cardiac tamponade in penetrating injuries leading to TCA. Consequently, RT remains the gold standard for TCA in cardiac tamponade, as it ensures effective release of the tamponade and enables both direct cardiac repair and definitive hemorrhage control [60]. A systematic review evaluating the role of pericardiocentesis in traumatic cardiac tamponade concluded that existing studies are limited and often biased towards survivors. The evidence suggests that pericardiocentesis may have a limited role in nontrauma centers where definitive surgical management is not immediately available; in such cases, it may serve as a temporary measure to facilitate safe transport to a higher level care facility [61].

#### 2.5. Which requirements must the material for performing RT meet?

We suggest that the surgical instrumentation for RT should be standardized, streamlined, robust and user-friendly. Ideally, all necessary components should be prepacked in a compact, purpose-built thoracotomy kit.

(Weak recommendation, very low-quality evidence)

##### Rationale

To date, only one study specifically evaluated materials required for RT. In a comparative animal model, a novel thoracic retractor facilitated a faster, easier thoracotomy than the conventional Finochietto model [62]. Beyond this, the specific requirements for RT instrumentation have primarily been described in narrative reviews [63, 64] or within the methods sections of clinical studies. These publications consistently advocate a standardized, streamlined, and robust set of instruments. Core components typically include a scalpel (No. 20 or 21), a thoracic retractor (Finochietto or Dubost-type), heavy-duty scissors (Mayo-type or plaster-of-Paris shears), vascular clamps, and forceps. These instruments are usually sterilely prepacked in compact, purpose-built thoracotomy kits to ensure rapid accessibility. Additional common components for approach, hemorrhage control, and definitive repair include Gigli saws (for sternotomy), suture materials, surgical staplers, and large-vessel clamps for aortic occlusion (such as DeBakey or Satinsky type clamps). The panel emphasizes that such prepacked kits minimize cognitive load and preparation time in high-stress scenarios.

### 3. Special considerations when performing RT in the prehospital setting

#### 3.1. Which specific safety measures should be taken into account when performing RT on scene?

As penetrating injuries are often caused by violent crimes, we suggest that RT should only be performed once scene safety has been ascertained.

(Best practice statement)

We suggest ensuring patient privacy by using visual shielding (e.g., privacy screens) and controlling scene access during the procedure.

(Best practice statement)

##### Rationale

No studies have so far evaluated specific scene safety protocols required before performing RT in the out-of-hospital setting. The panel, therefore, based its statements on clinical experience and sound reasoning. Given that penetrating injuries are frequently the result of violent crime, ensuring the safety of the patient, the emergency medical teams and other responders is paramount. In cases involving suspected or confirmed violence, scene safety must be definitively established by police or security personnel before medical teams approach the patient. Furthermore, when performing a highly invasive procedure, such as RT on scene, every effort must be made to preserve patient dignity. The use of visual shielding, such as privacy screens and the maintenance of a strictly controlled scene perimeter are essential and effective methods for protecting the patient from public view and ensuring an environment conducive to high-stakes medical intervention.

#### 3.2. Are blood products required to perform RT in the prehospital setting?

We suggest that blood products should be available in prehospital systems performing RT; however, their immediate availability is not a prerequisite to perform RT in the prehospital setting.

(Weak recommendation, very low-quality evidence)

##### Rationale

Currently, no direct evidence demonstrates that survival following on-scene RT is significantly influenced by the immediate availability of blood products in the prehospital setting. Transfusion of blood products in patients undergoing RT is common [65, 66]. A nationwide from the USA reported that patients who underwent RT because of penetrating trauma received a median of 8.0 (interquartile range, IQR 3.0–17.0) packed red blood cells [65]. A retrospective analysis of RT cases from a major US trauma center, however, found no independent association between survival to hospital discharge and the volume of packed red blood cells or platelets transfused [66]. Although RT can technically be performed without blood products, robust volume resuscitation remains a critical component of care. This necessity stems from the high prevalence of hemorrhagic shock among TCA patients eligible for RT and the fact that achieving return of spontaneous circulation often results in severe, uncontrolled bleeding. Given that early transfusion of blood products improves outcome in traumatic hemorrhagic shock [67, 68], several advanced EMS that have successfully integrated on-scene RT into their clinical practice now routinely carry blood products [16, 69, 70]. The panel acknowledges that, while blood products (e.g., packed red blood cells) are a valuable adjunct in the management of these patients, their absence should not preclude initiating potentially life-saving RT, particularly when the primary goal is relief of cardiac tamponade.

#### 3.3. Which requirements should the receiving hospital meet to provide care for patients after RT in the prehospital setting?

We recommend that hospitals receiving patients following prehospital RT must be major trauma centers with 24/7 thoracic or cardiac surgical capabilities.

(Best practice statement)

We suggest that both the receiving hospital and the EMS performing prehospital RT adhere to coordinated treatment and communication protocols

(Best practice statement)

##### Rationale

Reflecting the established practice of advanced EMS that have integrated prehospital RT [16, 69, 70] and in accordance with the panel’s position on institutional requirements for RT implementation, there was a strong consensus that receiving hospitals must be major trauma centers equipped with 24/7 thoracic or cardiac surgical capabilities. The management of TCA patients who achieve return of spontaneous circulation following RT is exceedingly complex. It necessitates immediate and comprehensive postresuscitation care, including massive transfusion protocols [65, 66] and, in all instances, timely definitive surgical repair of the underlying injuries. Consequently, it is essential that EMS providers and receiving trauma centers maintain strictly coordinated treatment protocols and robust communication channels. This ensures that the specialized trauma team, operating theater and surgical specialists are fully mobilized prior to the patient’s arrival, minimizing any delay in definitive care.

#### 3.4. How should the emergency control center be involved in the operational process of RT in the prehospital setting?

We suggest that the emergency control center serves as a critical link in the operational process of prehospital RT. Its role includes identifying eligible patients, rapidly dispatching and coordinating specialized resources, and facilitating early communication with the receiving hospital.

(Best practice statement)

##### Rationale

No scientific evidence has so far specifically evaluated the role of the dispatch center in the process of prehospital RT. Drawing on the established practice of advanced EMS [16] and their own clinical experience, the panel emphasizes that the dispatch center is a crucial component of the operational process. By enabling the early identification of eligible patients, rapidly dispatching specialized resources and facilitating early communication with receiving hospitals, the dispatch center can significantly minimize the time to intervention. Furthermore, proactive dispatch management ensures a coordinated, uninterrupted transition to postresuscitation care; however, the panel acknowledges that identifying TCA via telephone triage remains a significant challenge for dispatchers [71]. Consequently, the development and implementation of specific, high-sensitivity dispatch protocols for penetrating trauma and suspected TCA are essential to support the dispatcher’s role in the RT process.

#### 3.5. Should other emergency services be informed or trained about the RT process?

We suggest that other emergency organizations, particularly the police, should be briefed on the operational requirements of prehospital RT and encouraged to participate in joint simulations to optimize scene management and interagency coordination.

(Best practice statement)

##### Rationale

No scientific evidence has formally evaluated the necessity of interagency training regarding the RT process; however, as penetrating trauma is frequently the result of violent crime, law enforcement personnel are often first on scene and play a pivotal role in establishing and maintaining scene safety. Depending on the operational setting, various other emergency services may also be present when prehospital RT is initiated. In low-volume systems with a low prevalence of penetrating injuries, such as Austria, an advanced, highly invasive intervention like RT may be unfamiliar to nonmedical responders. To prevent coordination failures and to ensure a secure environment for the intervention, a shared understanding of the procedure’s necessity and operational requirements is essential. Consequently, the panel suggests that other emergency organizations, particularly the police, should be proactively engaged in multidisciplinary simulation training. Such joint exercises foster a mutual understanding of priorities, balancing life-saving medical interventions with scene security and forensic requirements, thereby optimizing the overall emergency response.

### 4. Education and training

#### 4.1. Which education and training are required to perform RT?

We suggest that physicians undergo a structured educational course including both cadaveric and multidisciplinary simulation training prior to performing RT.

(Weak recommendation, low-quality evidence)

We suggest that physicians trained in RT participate in periodic refresher training to maintain procedural skills and team coordination.

(Weak recommendation, low-quality evidence)

##### Rationale

The RT is a high-acuity, low-occurrence procedure that demands highly specialized technical and nontechnical skills from both the operator and the resuscitation team. Formal education and rigorous training are essential for acquiring and maintaining these competencies, particularly in low-volume systems. Research indicates that structured RT training programs are associated with improved adherence to strict indication criteria and survival rates at lower volume centers that are comparable to those at high-volume institutions [19, 72]. Both cadaveric models and multidisciplinary simulation training have proven to be effective methods for learning and maintaining the technical and nontechnical skills required for RT [73–75]. Although scientific data are lacking, a time-limited clinical rotation in a department of cardiac or thoracic surgery appears as another meaningful training option. Furthermore, a prospective observational study at a level I trauma center demonstrated that routine procedural training and integrated trauma simulations were associated with a significant reduction in the time required to successfully perform the intervention [76]. Given the rapid decline of surgical skills when not frequently practised, the panel emphasizes that initial competency-based training must be supplemented by regular, standardized refresher sessions to ensure procedural safety and efficiency.

#### 4.2. Should nonphysician healthcare professionals involved in the process of RT also undergo education and training?

We suggest that nonphysician healthcare professionals involved in the RT process should also undergo structured education and training. This training should focus on procedural assistance, equipment familiarity and coordinated team dynamics during RT.

(Best practice statement)

##### Rationale

There is no scientific evidence that specifically evaluates the educational requirements and training modalities for nonphysician healthcare professionals assisting with RT; however, given the complexity of both the surgical intervention and the clinical scenario, the panel unanimously agreed that all healthcare professionals involved in the RT process should also undergo structured education and training. These educational interventions should encompass the theoretical principles of RT, direct procedural and technical assistance, equipment management, crisis resource management, skills and scene management. In the experience of the panel, multidisciplinary training sessions integrating nonphysician staff, physicians and, for specific scenarios, nonmedical first responders such as the police, represent a vital strategy. Such joint simulations not only enhance technical proficiency but also optimize interagency communication and scene management, ensuring that every team member can function effectively under the high-pressure conditions of a traumatic cardiac arrest.

### 5. Quality management

#### 5.1. Which quality control measures should be taken when implementing RT?

We suggest that all systems performing RT implement comprehensive quality control and clinical governance measures. These should include mandatory structured case debriefings, regular clinical audits, and established feedback loops to ensure the ongoing refinement of protocols and training programs.

(Weak recommendation, very low-quality evidence)

##### Rationale

No studies have specifically evaluated which quality control measures are most effective for ensuring and maintaining optimized patient outcomes in healthcare systems that provide RT; however, systems that have successfully integrated prehospital RT into their clinical practice consistently report using rigorous quality control and medical governance programs. These typically include mandatory structured case debriefings, regular audits, and established feedback loops [16]. Such measures facilitate the rigorous monitoring of patient selection, procedural techniques, systemwide logistics, data governance and postprocedural care. Furthermore, they provide the necessary data to refine clinical protocols and training curricula. The panel emphasizes that in low-volume environments, each RT case represents a critical learning opportunity; therefore, a robust governance framework is essential to ensure procedural safety, ethical accountability, and continuous quality improvement.

#### 5.2. Which support options should be offered to healthcare professionals who performed or witnessed RT?

We suggest that systems performing RT provide easily accessible, low-threshold support mechanisms (e.g., peer support programs, professional psychological counselling) for all healthcare professionals who performed or witnessed RT.

(Best practice statement)

##### Rationale

While the systematic literature review identified no specific evidence regarding support options for healthcare professionals involved in RT, the panel unanimously agreed, based on expert consensus, that low-threshold support mechanisms must be integrated into any system performing RT. Given the invasiveness of the procedure and the high-stress scenarios in which it occurs, performing or witnessing RT can trigger significant posttraumatic stress, anxiety, and other adverse psychological reactions [77, 78]. This risk is particularly pronounced for healthcare providers in low-volume regions, where penetrating injuries and RTs are rare events. Institutional support frameworks, including peer support programs and access to professional psychological counselling, are proven to mitigate posttraumatic and moral distress following traumatic or adverse events [79, 80]. Proactive mental health support is therefore considered a fundamental component of institutional safety culture and staff retention in systems providing advanced trauma care.

### 6. Ethical and legal considerations

#### 6.1. Which ethical considerations should be taken into account when deciding on the indications for and performing RT?

We recommend that ethical decision-making must guide the indication of RT. This process must incorporate an objective assessment of survival probability, strict adherence to eligibility criteria, and a profound respect for patient dignity. Furthermore, it requires a balanced approach to resource allocation, transparent communication with families, and sensitivity to the emotional impact on both relatives and the healthcare team.

(Weak recommendation, low-quality evidence)

##### Rationale

The RT is an advanced resuscitative intervention that can facilitate survival in carefully selected TCA patients [16, 20, 32]; however, its maximum invasiveness raises profound ethical challenges, particularly in healthcare systems with a low prevalence of penetrating trauma. A primary ethical concern is “indication creep”, i.e., the risk of applying overly liberal criteria driven by a clinician’s inclination despite clear evidence of futility. Furthermore, the preservation of patient dignity is paramount given the inherent exposure of the procedure. Ethical deliberations also encompass provider safety (e.g., occupational exposure risks) and the principle of distributive justice, which requires responsible stewardship of limited resources such as blood products and intensive care unit capacity [81]. In the absence of scientific evidence to manage these complex dilemmas, the panel unanimously asserted that ethical principles must govern all RT-related decisions. In particular and comparable to any other emergency intervention, the decision to proceed with RT must strictly adhere to evidence-based selection criteria. Respect for patient dignity must be of crucial importance in all cases and at all times. Transparent communication with families and sensitivity to the emotional impact this procedure might have on both relatives and healthcare professionals are additional essential components of ethically responsible practice.

#### 6.2. Which legal considerations should be taken into account when performing RT?

We recommend that physicians performing RT adhere to the established legal framework and professional statutes governing life-saving emergency procedures in their respective jurisdiction.

(Best practice statement)

##### Rationale

Laws and regulations in most countries restrict the performance of surgical interventions, such as RT, to licensed medical doctors. Further jurisdiction-specific statutes (e.g., in emergency scenarios or when a thoracic or cardiac surgeon is unavailable) may apply and vary across countries. The panel, therefore, recommends that physicians performing RT adhere to the legal framework governing life-saving emergency procedures in their respective countries. The panellists acknowledge that strict adherence to evidence-based indications and surgical techniques, as well as accurate documentation, can further mitigate medicolegal risks [82, 83].

## Conclusion

This Delphi consensus, involving representatives from all major emergency medical societies in Austria, provides evidence-informed recommendations on system requirements, indications, procedural implementation, training, quality management, and ethical considerations for the systematic integration of RT. The statements underscore that carefully defined indications, structured multidisciplinary training, and robust system preparedness are essential to optimize patient outcomes. These consensus-based recommendations serve as a comprehensive framework for clinicians and policymakers to safely integrate this rare but potentially life-saving intervention into trauma systems characterized by a low prevalence of penetrating injuries.

## Supplementary Information

ESM1: Supplementary material 1

## Data Availability

Not applicable.
