# Supplementary material for: Resuscitative thoracotomy in traumatic cardiac arrest: Multisociety consensus recommendations for settings with a low prevalence of penetrating injuries
Source: Wien Klin Wochenschr. 2026 Jun 13;138(Suppl 7):623–40. doi: 10.1007/s00508-026-02771-3 (PMC13290978; doi:10.1007/s00508-026-02771-3)
Supplement: Supplementary file 1 — ESM1: Supplementary material 1 [file 508_2026_2771_MOESM1_ESM.docx]

*Address for correspondence:*

Priv.-Doz. Dr. Martin W. Dünser, DESA, EDIC; Austrian Society of Anaesthesiology, Reanimatology and Critical Care Medicine (ÖGARI), Vienna, Austria

Email: [martin.duenser@i-med.ac.at](mailto:martin.duenser@i-med.ac.at)

**Resuscitative Thoracotomy in Traumatic Cardiac Arrest: Multi-Society Consensus Recommendations from a Country with a Low Prevalence of Penetrating Injuries**

Martin W. Dünser, MD*^1,2^; Daniel Grassmann, MD*^3,4,5^; Thomas Hamp, MD^3^; Mario Krammel, MD^3,4,5^; Philipp Eller, MD^6,7^; Philip Eisenburger, MD^6,8^; Romana Erblich, MD^2^; Barbara Hallmann, MD^9,10^; Michael Halmich, LLM, SJD^11^; Klaus Hellwagner, MD, LLM^11,12^; Klaus Herbich, MD^3,13^; Harald Herkner, MD^14,15^; Igor Knez, MD^16,17^; Lukas L. Negrin, MD, PhD^18,19^; Marcel Rigaud, MD^9,20^; Joachim Schlieber, MD^21,22^; Sebastian Schnaubelt, MD, PhD^3,4,21^; Wolfgang Schreiber, MD^5,15^; Alexandra-Maria Stommel, MD^14,15^; Florian Tomaselli, MD^23,24^; Helmut Trimmel, MD^1,25^; Christoph Veigl, MD^4,26^; Wolfgang Voelckel, MD^13,22^; Stefan Watzka, MD^23,27^; Dominik Wiedemann, MD^16,28^; Paul Puchwein, MD^18,29^

*, both authors contributed equally

^1^, Austrian Society of Anaesthesiology, Reanimatology and Critical Care Medicine (ÖGARI), Vienna, Austria; ^2^, Department of Anaesthesiology and Critical Care Medicine, Johannes Kepler University Linz, Linz, Austria; ^3^, Emergency Medical Service Vienna (Berufsrettung Wien), Vienna, Austria; ^4^, Austrian Cardiac Arrest Awareness Association (PULS), Vienna, Austria; ^5^, Austrian Association of Emergency and Disaster Medicine (ÖNK); ^6^, Austrian Society of Internal and General Intensive Care and Emergency Medicine (ÖGIAIN); ^7^, Department of Internal Medicine, Medical University of Graz, Graz, Austria; ^8^, Department of Emergency Medicine and Internal Medicine, Klinik Floridsdorf, Vienna Healthcare Group, Vienna, Austria; ^9^, Working Group Emergency Medicine (AGN); ^10^, Department of Anaesthesiology and Intensive Care Medicine, Medical University of Graz, Graz, Austria; ^11^, Austrian Society of Ethics and Law in Emergency and Disaster Medicine (ÖGERN); ^12^, 7^th^ Department of Internal Medicine with Emergency Medicine, Klinik Hietzing, Vienna Healthcare Group, Vienna, Austria; ^13^, ÖAMTC Flugrettung, Vienna, Austria; ^14^, Austrian Association of Emergency Medicine (AAEM); ^15^, Department of Emergency Medicine, Medical University of Vienna, Vienna, Austria; ^16^, Austrian Society of Cardiac and Thoracic Vascular Surgery (ÖGHTG) (ÖGHTG); ^17^, Division of Cardiac Surgery, Department of Surgery, Medical University of Graz, Graz, Austria; ^18^, Austrian Society of Orthopaedics and Traumatology (ÖGOuT); ^19^, Department of Orthopaedics and Trauma Surgery, Medical University of Vienna, Vienna, Austria; ^20^, Department of Anaesthesiology and Critical Care Medicine, AUVA Trauma Centre Graz, Graz, Austria; ^21^, Austrian Resuscitation Council (ARC); ^22^, Department of Anaesthesiology and Intensive Care Medicine, AUVA Trauma Centre Salzburg, Salzburg, Austria; ^23^, Austrian Society of Thoracic Surgery (ÖGTC); ^24^, Department of Cardiac, Vascular and Thoracic Surgery, Johannes Kepler University Linz, Linz, Austria; ^25^, Karl Landsteiner Institute for Emergency Medicine and Patient Safety, Seebenstein, Austria; ^26^, Department of Anesthesiology and Intensive Care Medicine, University Hospital St. Pölten – NOE LGA, Karl Landsteiner University, St. Pölten, Austria; ^27^, Department of Thoracic Surgery, Klinik Floridsdorf, Vienna Healthcare Group, Vienna, Austria; ^28^, Department of Cardiac Surgery, University Hospital St. Pölten – NOE LGA, Karl Landsteiner University, St. Pölten, Austria; ^29^, Department of Orthopaedics and Traumatology, Medical University of Graz, Graz, Austria.

*Address for correspondence:*

Priv.Doz. Dr. Martin W. Dünser, DESA, EDIC; Austrian Society of Anaesthesiology, Reanimatology and Critical Care Medicine (ÖGARI), Vienna, Austria; Email: [martin.duenser@i-med.ac.at](mailto:martin.duenser@i-med.ac.at)

**Figure 1.** PRISMA Flow Chart of the systematic review of the literature.

**
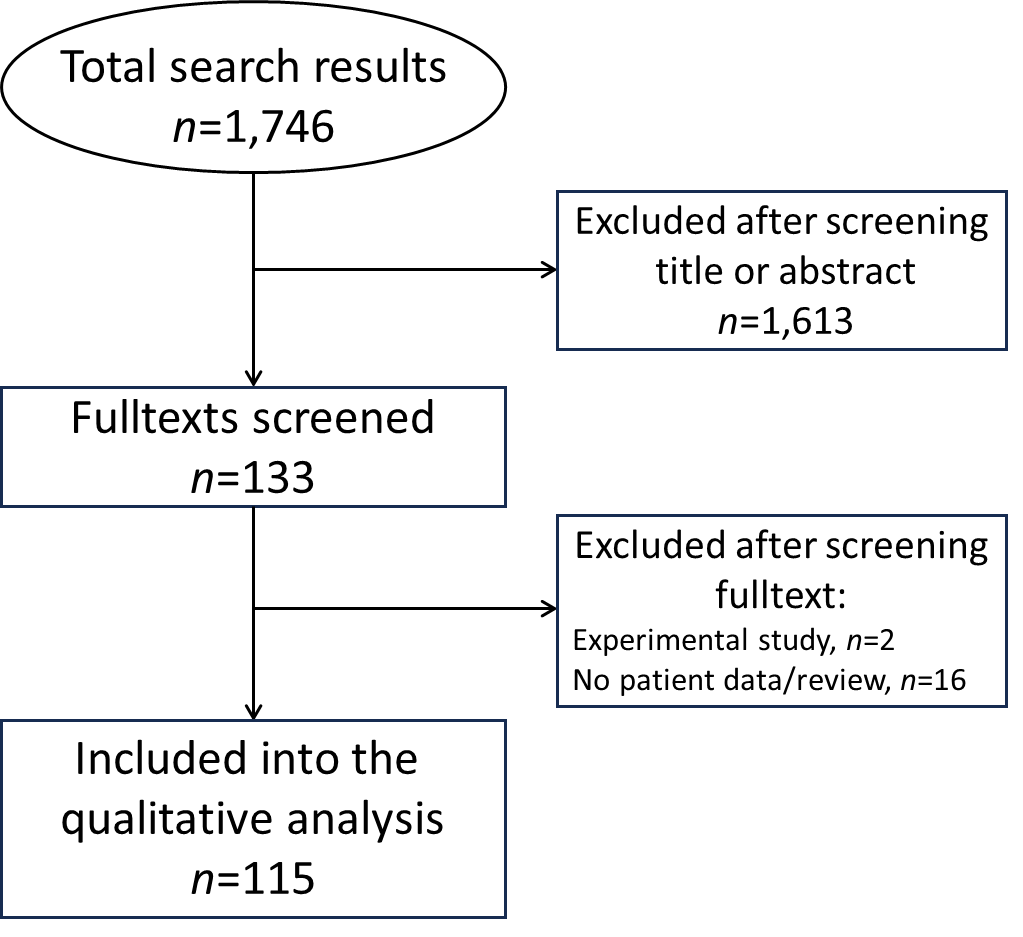
**

**Electronic Supplementary Material Table 1.** Methodology of the systematic review of the literature.

| **Aim** | to provide the expert panel with a comprehensive overview of the contemporary scientific evidence on resuscitative thoracotomy |
| --- | --- |
| **Type of publications considered** | randomized, controlled trials, prospective or retrospective cohort studies, case series, case reports, meta-analyses, guidelines/recommendations |
| **Publications excluded** | animal studies, reviews, opinion papers |
| **Patients** | Patients experiencing traumatic cardiac arrest as a direct consequence to trauma |
| **Age restrictions** | none |
| **Intervention** | resuscitative thoracotomy |
| **Information sources** | National Library of Medicine MEDLINE through PubMed® at https://pubmed.ncbi.nlm.nih.gov as well as hand search of reference lists and review articles |
| **Search terms** | “resuscitative thoracotomy” OR “clamshell thoracotomy” |
| **Publication dates** | January 1, 1966 until September 30, 2025 (subsequently extended to February 28, 2026) |
| **Language restriction** | English |
| **Number of reviewers** | two (RE, CV) |

**Electronic Supplementary Material Table 2.** The final set of questions to be answered by the expert panel.

1. **SYSTEM REQUIREMENTS FOR IMPLEMENTING RT**
   1. Which requirements must health systems meet to systematically implement RT?
2. **INDICATIONS FOR RT**
   1. What are the indications to perform RT?
   2. Does age or functional status influence the indication to perform RT?
   3. Is point-of-care ultrasound required before performing RT?
   4. Should RT also be performed in pregnant women?
   5. Which factors need to be considered when indicating RT in case of multiple casualties?
3. **PRACTICAL IMPLEMENTATION OF RT**
   1. Which human resources are required to perform RT?
   2. Who should perform RT in the absence of a thoracic or cardiac surgeon?
   3. Which technique should be used to perform RT?
   4. Is pericardiocentesis an alternative to RT in patients with traumatic cardiac tamponade?
   5. Which requirements must the material for performing RT meet?
4. **SPECIAL CONSIDERATIONS WHEN PERFORMING RT IN THE PRE-HOSPITAL SETTING**
   1. Which specific safety measures should be taken into account when performing RT on scene?
   2. Are blood products required to perform RT in the pre-hospital setting?
   3. Which requirements should the receiving hospital meet to provide care for patients after RT in the pre-hospital setting?
   4. How should the emergency control centre be involved in the operational process of RT in the pre-hospital setting?
   5. Should other emergency services be informed or trained about the RT process?
5. **EDUCATION AND TRAINING**
   1. Which education and training are required to perform RT?
   2. Should non-physician healthcare professionals involved in the process of RT also undergo education and training?
6. **QUALITY MANAGEMENT**
   1. Which quality control measures should be taken when implementing RT?
   2. Which support options should be offered to healthcare professionals who performed or witnessed RT?
7. **ETHICAL AND LEGAL CONSIDERATIONS**
   1. Which ethical considerations should be taken into account when deciding on the indication for and performing RT?
   2. Which legal considerations should be taken into account when performing RT?
